# Dataset on the effect of Benzene exposure on genetic damage, hematotoxicity, telomere length and polymorphisms in metabolic and DNA repair genes

**DOI:** 10.1016/j.dib.2020.105869

**Published:** 2020-06-18

**Authors:** Jing-chao Ren, Huan Liu, Guang-hui Zhang, Tongshuai Wang, Jingzhi Li, Tingting Dong, Hantian Wu, Zhao-lin Xia

**Affiliations:** a School of Public Health, Xinxiang Medical University, 601 Jinsui Road, Xinxiang, 453003, China; b Department of Occupational Health and Toxicology, School of Public Health, Fudan University, 138 Yixueyuan Road, Shanghai, 200032, China

**Keywords:** Benzene, Telomere length, Genetic damage, Micronucleus frequency, Hematotoxicity, Metabolic genes, DNA repair genes, Polymorphism

## Abstract

In this paper, we present an occupational dataset to evaluate benzene exposure on the effective biomarkers of genetic damage, indicated as cytokinesis-block micronucleus (MN) frequency, hematotoxicity, indicated as white blood cells (WBC) counts, and molecular marker of telomere length (TL). And we further to eliminate the mechanism of benzene induced damage. Then evaluate the effects of sites polymorphism in environmental response genes, including 18 sites in metabolic and DNA repair genes, and the interaction between gene polymorphism and benzene exposure. This dataset is supplementary to the submitted research by [1] focused on the biomarkers TL, and a detailed description of the subjects sampling, biomarkers detection, data analysis and discussion are discussed in detail.

Specifications Table

**Table:** 

Subject	Public Health and Health Policy
Specific subject area	Environmental and occupational health
Type of data	Table Figure
How data were acquired	The volunteers were recruited during occupational physical examination. We conducted experiment of cytokinesis-block micronucleus frequency, white blood cells (WBC), telomere length, and 18 polymorphic sites in environmental response genes, including metabolic and DNA repair genes.
Data format	Raw and analyzed
Parameters for data collection	Micronucleus frequency, White blood cells counts, Rlative telomere length, genotype of polymorphism genes
Description of data collection	During the physical examination, we propagated our scientific program to the workers. If the workers volunteer to participate, they should sign an ‘informed consent’. Then, we took part in the collection of basic information and blood samples for testing.
Data source location	Institution: Fudan Univeristy City/Town/Region: Wenzhou; ouhai district; Zhejiang Country: China
Data accessibility	Raw data are provided with this article as supplementary material.
Related research article	Jing-chao Ren, Huan Liu, Guang-hui Zhang, Tongshuai Wang, Jingzhi Li , Tingting Dong, Hantian Wu, Zhao-lin Xia. Interaction effects of environmental response gene polymorphisms and benzene exposure on telomere length in shoe-making workers. Chemosphere, In press.

Value of the Data

• This data provides biomarkers of genetic damage indicated as cytokinesis-block micronucleus frequency, and hematotoxicity, indicated as white blood cells counts in benzene exposed workers.
• This data also provides demographic characteristics and telomere length of benzene exposed workers and controls.
• This data presents 18 environmental response gene polymorphisms, including metabolic and DNA repair genes.

## 1 Data

**Fig. 1:**
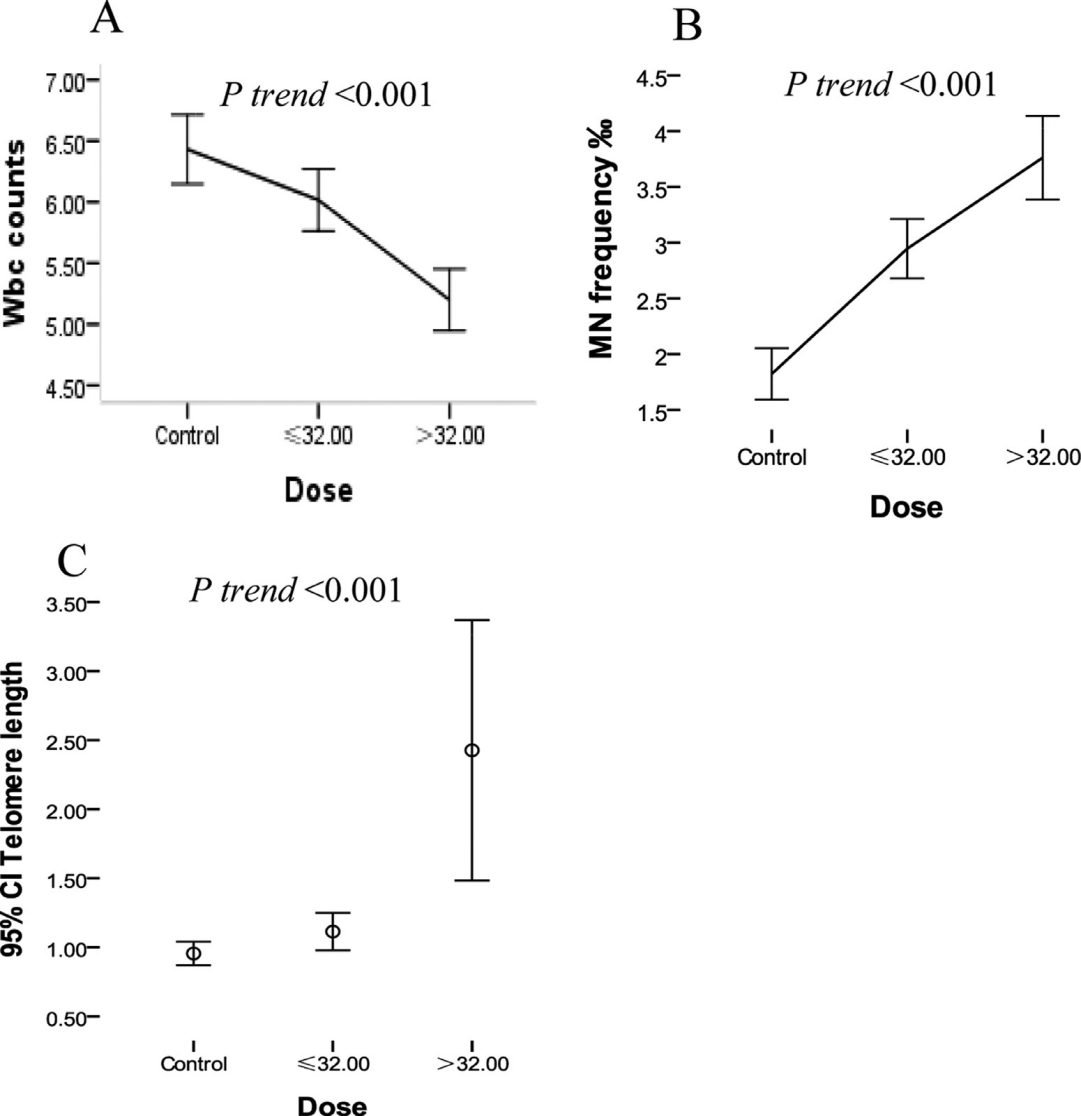
Analyzed data of dose response relationship between biomarkers and benzene exposure CED (mg/m^3^-year). The x-axis represents the cumulative exposure dose (CED) of benzene exposure. A showed the effects of benzene exposure on white blood cell count (WBC). B presented the effects of benzene exposure on micronucleus (MN) frequency. C showed the effects of benzene exposure on telomere length (TL).

In this paper, we provide a benzene exposure workers recruited in shoes plant in Wenzhou, China. The dataset provide the analysis of benzene exposure on effects of biomarkers of peripheral blood cytokinesis-block micronucleus frequency, white blood cells (WBC), telomere length, and the polymorphic sites in environmental response genes, including metabolic and DNA repair genes. Fig. 1 showed analyzed data on dose response relationship between effect biomarkers and benzene exposure cumulative exposure dose (CED) (mg/m^3^-year). The raw data are provided with this article as supplementary material.

Table 1 presented the analyzed data effects of gene polymorphisms and benzene exposure on peripheral blood white blood cell (WBC). Multi-linear regression indicated that rs13181 in XPD Lys751Gln, null GSTM1, and rs 3,813,867 in CYP2E1 were associated with reduced WBC. Rs 3,212,986 in ERCC1 3′-UTR G8092T, TG genotype was associated with a increased WBC.

**Table 1:** The analyzed data on effects of gene polymorphisms and benzene exposure on White blood cell. .

Variables	*Β (95%CI)*	T	*P*
CED (mg/m^3^-year)^a^	−0.004(−0.006, −0.002)	15.11	<0.001
Rs 13,181			
TG (versus TT)	−0.24(−0.77,0.29)	0.78	0.376
GG (versus TT)	−1.74(−3.27,−0.21)	4.99	0.026
Rs 3,212,986			
TG (versus GG)	0.62(0.23,1.01)	9.88	0.002
TT (versus GG)	0.40(−0.12,0.92)	2.24	0.134
GSTM1			
Null versus Non-null	−0.40(−0.76, −0.04)	4.64	0.031
Rs 3,813,867	−0.44(−0.85, 0.02)	4.27	0.039
GC (versus GG)	−0.52(−0.91, −0.12)	6.51	0.011
CC (versus GG)	−0.31(−1.57, 0.96)	0.23	0.634

The Multiple linear analysis was conducted to analyze the gene polymorphisms on white blood cell in separate models, after adjusting gender, age, smoking and alcohol using.

a CED, cumulated exposed dose, which was continuous variable.

## 2 Experimental design, materials and methods

### 2.1 Subjects

In total, 294 benzene-exposed participants, ranging from 17 to 57 years old, were enrolled in 2011 from Wenzhou, China. And a control group consisting of 102 indoor workers, were selected in the same year from the same city. We predicted the number of samples before the research for MN frequency, TL and genetic polymorphism.

#### 2.1.1 For predict Tl sample number

The formula of sampling numbers comparing the difference of two samples was:

$$n1=n2={\left[\frac{(z_{\alpha /2}+z_{\beta /2})\sigma }{\delta }\right]}^{2}$$

Significant level α = 0.05, power of test 1–β = 0.9, ‘δ’ was the mean value of difference between benzene exposed group and control group. According to previous reports [2], δ = TL_exposure_-TL_control_ =1.37–1.26=0.11, the largest *S* = 0.23, and n1 = n2 = 90. Therefore, at least 90 benzene exposed workers and controls were needed in the data.

#### 2.1.2 For predict sample number of polymorphic form of metabolic genes and DNA repair genes

According to pre-experiment, the mutant allele (the Heterozygote + Homozygous mutant) frequency was 20%. The equation was:

$$N={\left[\frac{\left(z_{\alpha /2}+z_{\beta /2}\right)\sigma }{\delta }\right]}^{2}\left({Q_{1}}^{-1}+{Q_{2}}^{-1}\right)$$

If the ‘δ’, the difference of TL between the wild type workers and mutant workers was postulated as 0.15, 154 benzene-exposed workers were needed. Generally speaking, larger the sample size, better will be the results for polymorphic form of data. Our sample size can detect the TL difference of 0.15.

#### 2.1.3 For predict MN frequency

Micronucleus (MN) frequency suit for Poisson distribution, and the formula of sampling numbers comparing the rata of two samples were as follows:

$$n1=n2={\left(u_{\alpha }+u_{\beta }\right)}^{2}\left(\lambda_{1}+\lambda_{2}\right)/{\left(\lambda_{1}-\lambda_{2}\right)}^{2}$$

Where λ_1_ and λ_2_ are mean of the control and exposed workers, respectively. If we postulated that α = 0.05, β = 0.10. According to previous data [3], λ_1_ and λ_2_ were 7.06 and 4.83. We can deduce the sampling numbers were 21 for exposed and control separately. According to previous data of our team [4], with the lowest MN frequency in the exposed workers, 44 numbers of the exposed and control were needed in the dataset.

### 2.2 Genotyping SNPs in DNA repair and metabolic genes

In total, 18 polymorphisms in DNA repair and metabolic genes were conducted in data. Polymorphisms of GSTM1, GSTT1, GSTP1 (rs1695), CYP2E1 (rs3813867, rs2031920, rs6413432), mEH exon 3 (rs1051740), and mEH exon 4 (rs2234922) were detected by restriction fragment length polymorphism (RFLP) analysis, the detailed methods were reported in previous report [5]. For BER pathway: XRCC1 (rs25489, rs25487) and APE1(rs1130409); and NER pathway: ERCC1 (rs3212986), XPA (rs1800975), XPC (rs2228000, rs2228002), XPD (rs13181, rs1799793), and XPG (rs17655) detection, detailed primers were designed and supplied by Xiangyin Biotechnology Co (Shanghai, China) with method of kompetitive allele specific PCR (KASP), presented in Table 2.

**Table 2:** Primers and sequence for DNA repair genes.

ID	Primer_Allele FAM	Primer_Allele HEX	Primer_Common	Sequence
rs1800975	AGGTCCTCGGAGTGGGCCA	GGTCCTCGGAGTGGGCCG	CCCGTCGGCCGCCGCCAT	CTAGGTCCTCGGAGTGGGCC[A/G]GAGATGGCGGCGGCCGACGG
rs25489	CCAGTGCCAGCTCCAACTCA	CCAGTGCCAGCTCCAACTCG	CTGGGGCTGTGGCTGGGGTA	TCCAGTGCCAGCTCCAACTC[A/G]TACCCCAGCCACAGCCCCAG
rs1130409	AATTCTGTTTCATTTCTATA- GGCGAG	GCTAATTCTGTTTCATTTCT- ATAGGCGAT	CACAATCACCCGGCCTTCCTGAT	TGTTTCATTTCTATAGGCGA[G/T]GAGGAGCATGATCAGGAAGG
rs3212986	CAGGCTGCTGCTGCTGCTG	CACAGGCTGCTGCTGCTGCTT	AGGCCGGGACAAGAAGCGGAA	CACAGGCTGCTGCTGCTGCT[G/T]CTTCCGCTTCTTGTCCCGGC
rs17655	ACATTCATTAAAGATGAA- CTTTCAGCATC	ACATTCATTAAAGATGAACTT- TCAGCATG	CAGAATCATCTGATGGATCTT- CAAGTGAA	TAAAGATGAACTTTCAGCAT[C/G]TTCACTTGAAGATCCATCAG
rs13181	AGAATCAGAGGAGACGCTGA	CTAGAATCAGAGGAGACGCTGC	AGAGCTGCTGAGCAATCTGCTCTAT	AGCAATCTGCTCTATCCTCT[T/G]CAGCGTCTCCTCTGATTCTA
rs1799793	CACCCTGCAGCACTTCGTT	CTCACCCTGCAGCACTTCGTC	CGGACGCCCACCTGGCCAA	TGGCCAACCCCGTGCTGCCC[A/G]ACGAAGTGCTGCAGGGTGAG
rs25487	CGGCGGCTGCCCTCCCA	GGCGGCTGCCCTCCCG	AGGGTTGGCGTGTGAGGCCTTA	GCGTCGGCGGCTGCCCTCCC[A/G]GAGGTAAGGCCTCACACGCC
rs2228000	TGAAGAGCTTGAGGATGCCG	GCTTGAAGAGCTTGAGGATGCCA	GAGCCATCGTAAGGACCCAAGCTT	TAAGGACCCAAGCTTGCCAG[C/T]GGCATCCTCAAGCTCTTCAA
rs2228001	GGGCGCTCAGCTCACAGCTT	GGCGCTCAGCTCACAGCTG	GCAGCTTCCCACCTGTTCCCAT	CCCACCTGTTCCCATTTGAG[A/C]AGCTGTGAGCTGAGCGCCCA

The KASP genotyping platform requires two different, allele-specific, competing forward primers, one labeled with FAM, and the other forward labeled with HEX, and one common reverse primer were needed (table 2). The last column was the sequence of polymorphism sites and its mutant alleles.

## Declaration of Competing Interest

The authors declared that they have no conflicts of interest to this work.

We declare that we do not have any commercial or associative interest that represents a conflict of interest in connection with the work submitted.

## Appendix Supplementary materials

Image, application 1

Image, application 2

Image, application 3

Image, application 4

## References

[1] Ren Jing-chao, Liu Huan, Zhang Guang-hui, Wang Tongshuai, Li Jingzhi, Dong Tingting, Wu Hantian, Xia Zhao-lin (2020). Interaction effects of environmental response gene polymorphisms and benzene exposure on telomere length in shoe-making workers. Chemosphere.

[2] Bassig B.A., Zhang L., Cawthon R.M., Smith M.T., Yin S., Li G., Hu W., Shen M., Rappaport S., Barone-Adesi F., Rothman N., Vermeulen R., Lan Q. (2014). Alterations in leukocyte telomere length in workers occupationally exposed to benzene. Environ. Mol. Mutagen..

[3] Maffei F., Hrelia P., Angelini S., Carbone F., Cantelli F.G., Barbieri A., Sanguinetti G., Mattioli S., Violante F.S. (2005). Effects of environmental benzene: micronucleus frequencies and haematological values in traffic police working in an urban area. Mutat. Res..

[4] Yang B.Y., Lv J.P., Cheng W., Zhou L., F. Ye, Y J., Sun P., Feng N., N. Wang, Q. Jin, R F., Sun P., Cheng Z., X. Xia, Z L. (2012). Micronucleus occurrence in Chinese workers occupationally exposed to benzene. Eur. J. Oncol..

[5] Zhang G.H., Ye L.L., Wang J.W., Ren J.C., Xu X.W., Feng N.N., Zhou L.F., Ru J.G., Hao Y.H., Tian W., Sun P., Au W.W., Christiani D.C., Xia Z.L (2014). Effect of polymorphic metabolizing genes on micronucleus frequencies among benzene-exposed shoe workers in China. Int. J. Hyg. Environ. Health.

