# Supplementary material for: Dataset on the effect of Benzene exposure on genetic damage, hematotoxicity, telomere length and polymorphisms in metabolic and DNA repair genes
Source: Data Brief. 2020 Jun 18;31:105869. doi: 10.1016/j.dib.2020.105869 (PMC7327812; doi:10.1016/j.dib.2020.105869)
Supplement: Supplementary file 1 [file mmc1.docx]

外周血采样知情同意书

**项目负责人：**复旦大学公共卫生学院 夏昭林,张光辉

**电话**： 18237376831

**1. 研究简介和研究目的**

化学致癌物职业接触人群早期遗传损伤检测与风险评定**课题组邀请您作为苯作业工人和对照组参加。检测外周血遗传损伤、甲基化改变和差异蛋白。检测尿样中苯的代谢产物。**本研究由国家自然科学委员会资助。我们希望本研究的信息会对随后开展有效的**环境**致遗传损伤的预防项目或制定相关政策有所帮助。

**如果同意参加，您将被要求完成调查后在调查表上签字表示同意。**

**3. 研究过程**

您将参加约数分钟的**问卷调查**，调查一般信息如姓名、年龄、性别等，行为信息如吸烟、饮酒等信息。在职业卫生健康体检的同时进行采血，**采血样3ml**。我们委托体检医院进行外周血采血，确保安全。

**4. 可能的风险--**个人信息泄露和采血安全

将要求每位**参加研究的人员签署保密协定**，以**保证研究人员不会泄露你的有关信息**。此次采血是和健康体检同时进行，**保证严格操作，确保安全。**

**5. 可能的受益**

参加本项目研究将对您的健康状况得到适当评价，可以及时发现一些早期健康损害。

**6. 研究记录的保密**

所有研究信息将绝对保密，只有项目研究人员才能接触到这些信息。

**7. 研究对象的权利**

你可以在任何时间退出本研究而不会有任何后果。参加本研究也不会损害到你已有的任何权利。你可与复旦大学公共卫生学院伦理委员会联系，电话021-54237051，这个机构代表你的利益。

**被调查者（签名）**：__________________

**调查员**：___________________ 日期__________________
