# Supplementary material for: Dataset on the effect of Benzene exposure on genetic damage, hematotoxicity, telomere length and polymorphisms in metabolic and DNA repair genes
Source: Data Brief. 2020 Jun 18;31:105869. doi: 10.1016/j.dib.2020.105869 (PMC7327812; doi:10.1016/j.dib.2020.105869)
Supplement: Supplementary file 2 [file mmc2.docx]

Informed consent of peripheral blood sampling

**Project Leader:** Zhao-lin Xia, Guang-hui Zhang, School of public health, Fudan University

**Phone** ： 18237376831

**1. Introduction and the purpose of study**

The research group of early genetic damage detection and risk assessment of occupational exposure to chemical carcinogens, **invites you to participate as benzene workers and control group. Peripheral blood genetic damage, methylation change and differential protein were detected.** The metabolites of benzene in urine were detected. This research is supported by the National Natural Science Commission. We hope that the information of this study will be helpful to carry out effective prevention projects or formulate relevant policies.

**If you agree to participate, you will be needed to sign the name before completing the survey.**

**3. Study program**

You will take part in **a questionnaire survey** for several minutes, which will survey general information such as name, age, gender, etc., and behavioral information such as smoking, drinking, etc. At the same time of occupational health examination, **3ml blood** samples were collected. We entrust physical examination hospital to collect blood from peripheral blood to ensure safety.

**4. Possible risks --** personal information disclosure and blood sampling security

**Each participant will be required to sign a confidentiality agreement to ensure that the researcher does not disclose your relevant information.** The blood collection is carried out **at the same time with the health examination to ensure strict operation and safety.**

**5. Possible benefits**

Taking part in this project research will get a proper evaluation of your health status, and you can find some early health damage in time.

**6. Confidentiality of research records**

All research information will be kept absolutely confidential and only project researchers will have access to it.

**7. Rights of subjects**

You can quit the study at any time without any consequences. Participation in this study will not prejudice any of your existing rights. You can contact the ethics committee of School of public health, Fudan University, at 021-54237051, which represents your interests.

**Volunteer (signature)**：__________________

**Investigator:**___________________ time_________________
