# Supplementary material for: Dataset on the effect of Benzene exposure on genetic damage, hematotoxicity, telomere length and polymorphisms in metabolic and DNA repair genes
Source: Data Brief. 2020 Jun 18;31:105869. doi: 10.1016/j.dib.2020.105869 (PMC7327812; doi:10.1016/j.dib.2020.105869)
Supplement: Supplementary file 3 [file mmc3.docx]

健康状况调查表

工 号： 血样编号：

单位名称： 市 （县）_____________ 车 间：______________

1. 姓名：________________2.性别：①男 ②女

2. 身高：________cm， 体重：________kg. 4. 民族：____族 籍贯：______省（市）____市（县）

3. 文化程度： ①小学及以下 ②初中 ③高中 ④大专及大专以上

4. 出生年月：______年______月

5. 接触苯工龄：________年；日工作时间：________小时

6. 您在该厂的工作情况（若未换过工作车间，只填一个车间；若换过车间，请按次序填写）

（1）工作车间___ ____岗位____________起止年月_______年_____月至_____年____月

（2）工作车间___ ____岗位____________起止年月_______年_____月至_____年____月

7. 防护用品的使用情况： ①无 ②偶尔 ③经常

8．下班后是否洗澡： ①无 ②偶尔 ③经常

9. 在车间是否吸烟： ①无 ②偶尔 ③经常

10. 在车间是否吃东西： ①无 ②偶尔 ③经常

11．上下班是否换工作衣： ①无 ②偶尔 ③经常

12．近2周有无接受x光照射：①无 ②有， 几次___

13．吸烟情况：①不吸 ②<5支/天 ③5--10支/天 ④≥10支/天 ⑤戒烟

14. 喝酒情况： ①从不 ②≤2次/月 ③2-4次/月 ④2-4次/周 ⑤≥5次/周

15所喝酒的种类: ①白酒 ②黄酒 ③葡萄酒 ④啤酒

16. 既往有无疾病史？ ①无 ②有 ，是何种疾病？____________________________

17. 家族中有其他成员也患有该疾病吗？ ①无 ②有

18. 家族中有其他遗传性疾病吗？ ①无 ②有 ，是何种疾病？＿＿＿＿

19. 您有无肝炎病史？ ①无 ②有 ，是哪种类型？＿＿＿＿＿＿

20. 您有没有以下症状： （1）失眠多梦 ①无 ②有时 ③经常

（2）注意力不集中 ①无 ②有时 ③经常

（3）记忆力减退 ①无 ②有时 ③经常

（4）乏力 ①无 ②有时 ③经常

（5）头晕 ①无 ②有时 ③经常

21.您有没有以下症状: （1）牙龈出血 ①无 ②有时 ③经常

（2）鼻腔出血 ①无 ②有时 ③经常

（3）皮下瘀斑 ①无 ②有时 ③经常

（4）月经异常 ①无 ②有时 ③经常

调查和采血经过知情同意（签字）：＿＿＿

调查员： 日期：2011年＿＿月＿＿
