# Supplementary material for: Dataset on the effect of Benzene exposure on genetic damage, hematotoxicity, telomere length and polymorphisms in metabolic and DNA repair genes
Source: Data Brief. 2020 Jun 18;31:105869. doi: 10.1016/j.dib.2020.105869 (PMC7327812; doi:10.1016/j.dib.2020.105869)
Supplement: Supplementary file 4 [file mmc4.docx]

Health status questionnaire

NUMBER： Blood sample No：

Company name： City_____________ Workshop ：______________

1. Name：________________2.Sex：①Male ②Female

2. Height：________cm，Weight：________kg. 4. Ethics：____ Birth place：______City____Country

3. education： ①Primary school and below ②junior middle school ③high school ④Junior college or above

4. birth data：______Year______Month

5. Exposure to benzene duration：________years；work hours a day：_______hours

6. Your work situation in the factory (if you have not changed the workshop, only one workshop will be filled in; if you have changed the workshop, please fill in in order)

（1）Workshop ___ ___Position_______ from ______years_____month, to_____ years_____month

（2）Workshop ___ ___Position_______ from ______years_____month, to_____ years_____month

7. Use of protective equipment：① No ② occasionally ③ often

8．Shower after work： ① No ② occasionally ③ often

9. Do you smoke in the workshop： ① No ② occasionally ③ often

10. Whether to eat in the workshop： ① No ② occasionally ③ often

11．Whether to change work clothes when going to work or not： ① No ② occasionally ③ often

12．X-ray exposure in recent 2 weeks：①n0 ②yes， how much ___

13．Smoking：① No smoking ② < 5 / day ③ 5 -- 10 / day ④ ≥ 10 / day ⑤ quit smoking

14. Alcohol using：① Never ② ≤ 2 times / month ③ 2-4 times / month ④ 2-4 times / week ⑤ ≥ 5 times / week

15 Types of drinking: ① White wine ② yellow wine ③ wine ④ beer

16. Have you ever had a history of disease? ① No ② yes What kind of disease is it?______

17. Is there any other member of the family suffering from the disease? ① No ② yes

18. Are there any other genetic diseases in the family? ① No ② yes What kind of disease is it?＿＿＿＿

19. Do you have a history of hepatitis? ① No ② yes, what type?＿＿＿＿＿＿

20. Do you have the following symptoms: (1) insomnia and dreaminess ① no ② sometimes ③ often

(2) Inattention ① no ② sometimes ③ often

(3) Memory loss ① no ② sometimes ③ often

(4) Fatigue ① no ② sometimes ③ often

(5) Dizziness ① no ② sometimes ③ often

21. Do you have the following symptoms: (1) gingival bleeding ① no ② sometimes ③ often

(2) Nosebleed ① no ② sometimes ③ often

(3) Subcutaneous ecchymosis ① no ② sometimes ③ often

(4) Abnormal menstruation① no ② sometimes ③ often

After informed consent (signature) for investigation and blood collection:

Investigator:

Date:
